# Oil Family Typing Using a Hybrid Model of Self-Organizing Maps and Artificial Neural Networks

**DOI:** 10.1021/acsomega.1c05811

**Published:** 2022-04-02

**Authors:** Majid Safaei-Farouji, Shahab S. Band, Amir Mosavi

**Affiliations:** † School of Geology, College of Science, University of Tehran 1417935840 Tehran, Iran; ‡ Future Technology Research Center, College of Future, National Yunlin University of Science and Technology, 123 10 University Road, Section 3, Douliou, Yunlin 64002, Taiwan, ROC; § John von Neumann Faculty of Informatics, Obuda University, 1034 Budapest, Hungary; ∥ Institute of Information Society, University of Public Service, 1083 Budapest, Hungary; ⊥ Institute of Information Engineering, Automation and Mathematics, Slovak University of Technology, 812 37 Bratislava, Slovakia

## Abstract

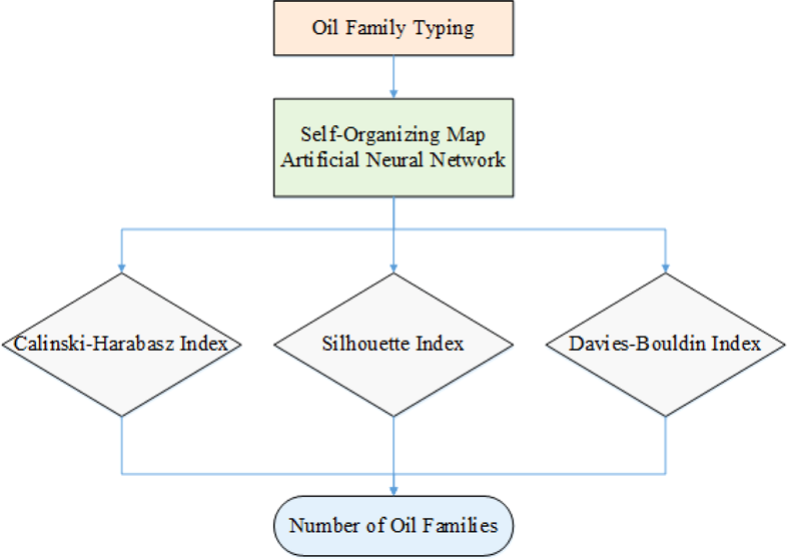

Identifying the number of oil families in petroleum basins provides practical and valuable information in petroleum geochemistry studies from exploration to development. Oil family grouping helps us track migration pathways, identify the number of active source rock(s), and examine the reservoir continuity. To date, almost in all oil family typing studies, common statistical methods such as principal component analysis (PCA) and hierarchical clustering analysis (HCA) have been used. However, there is no publication regarding using artificial neural networks (ANNs) for examining the oil families in petroleum basins. Hence, oil family typing requires novel and not overused and common techniques. This paper is the first report of oil family typing using ANNs as robust computational methods. To this end, a self-organization map (SOM) neural network associated with three clustering validity indexes was employed on oil samples belonging to the Iranian part of the Persian Gulf oilfields. For the SOM network, at first, 10 default clusters were selected. Afterward, three effective clustering validity coefficients, namely, Calinski–Harabasz (CH), Silhouette (SH), and Davies–Bouldin (DB), were studied to find the optimum number of clusters. Accordingly, among 10 default clusters, the maximum CH (62) and SH (0.58) were acquired for 4 clusters. Similarly, the lowest DB (0.8) was obtained for four clusters. Thus, all three validation coefficients introduced four clusters as the optimum number of clusters or oil families. According to the geochemical parameters, it can be deduced that the corresponding source rocks of four oil families have been deposited in a marine carbonate depositional environment under dysoxic–anoxic conditions. However, oil families show some differences based on geochemical data. The number of oil families identified in the present report is consistent with those previously reported by other researchers in the same study area. However, the techniques used in the present paper, which have not been implemented so far, can be introduced as more straightforward for clustering purposes in oil family typing than those of common and overused methods of PCA and HCA.

## 1 Introduction

Identifying the relationship between oil samples and grouping them, known as oil family classification, as a part of petroleum system studies, plays a paramount role in various aspects of the oil industry, including exploration, development, and so forth. The primary outcomes of oil family typing are detecting migration pathways and evaluating the continuity between different oil reservoirs.^1^

In the following introduction, the method used and recent works are generally explained. The second part of the paper is devoted to the data preparation and methodology. Then, the obtained results are discussed in the third section. Ultimately, the final part of the study provides a summary of the findings.

It is for a long time that geochemists have used the statistical techniques principal component analysis (PCA) and hierarchical clustering analysis (HCA) to group oil families in petroleum basins.^2,3^ However, it is an undeniable fact that artificial intelligence (AI) and machine learning (ML) systems are developing on a regular basis and provide various applications for scientists,^4−8^ and petroleum geochemists are no exception. AI and ML techniques in petroleum-related studies have been widely used in recent years. Hemmati-Sarapardeh et al.^12^ conducted the modeling natural gas compressibility using a kind of artificial neural network (ANN). Amooie et al.^13^ took advantage of ML methods for geological carbon storage studies. Bolandi et al.^15^ evaluated source rock characteristics using ML methods. Bolandi et al.^16^ studied the organic facies of source rocks by combining ML and ANNs. Tabatabaei et al.^17^ utilized the ML algorithm for the estimation of total organic carbon (TOC) from well log data. Kadkhodaie-Ilkhchi et al.^19^ integrated individual smart ML models with a committee machine intelligent system to approximate TOC from petrophysical well logs. Ghiasi-Freez et al.^20^ used committee machines to predict permeability from petrographic image analysis. Amiri-Ramsheh et al.^25^ conducted a study about modeling of wax disappearance temperature using different AI and ML methods. In addition to the mentioned studies, recently researchers have used AI and ML for organic geochemistry purposes. For example, Safaei-Farouji and Kadkhodaie^30^ used intelligent AI and ML methods for the estimation of kerogen type from petrophysical well logs. Collectively, even though AI and ML methods have been used in various petroleum-related fields, oil family typing using an ANN is missing. ANNs have various applications, one of which is clustering.^31−33^ Therefore, oil family grouping as a kind of clustering problem can be solved via ANNs.

The self-organization map (SOM) function as an ANN proposed by ref (34) maps multidimensional data to a two-dimensional space. This space is created with the help of a competitive and unsupervised learning process. The SOM neural network preserves the topological properties of the input space by utilizing a neighborhood function. Actually, the resulting map illustrates the relationship between input patterns.^35,36^

The primary use of SOM is clustering and other types of unsupervised classifications.^35,36^ So far, for oil family grouping, limited common statistical methods, such as PCA and HCA, have been used, but using ANNs is entirely missing. Rabbani et al.^2^ geochemically analyzed 33 oil samples from several oil fields in the Persian Gulf’s Iranian sector. They defined four main oil families using the statistical methods of PCA and HCA. Mashhadi and Rabbani^37^ also geochemically investigated 20 oil samples from oil fields in the Iranian part of the Persian Gulf. They identified two distinct genetic oil families using PCA. In another study, Hosseini et al.,^3^ based on the study of 14 oil samples from the eastern Iranian sector of the Persian Gulf and implementing HCA, identified two different oil families.

Petroleum geochemistry studies of the examined area have been conducted by previous research studies;^2,3,37^ correspondingly, in the present paper, we focus on using an SOM neural network as a novel paradigm to determine oil families in the region. Indeed, the present study enables us to relate our outcomes to previously published works in the study area while using more database and introducing a new method for oil family typing.

## 2 Materials and Methods

**Figure 1:**
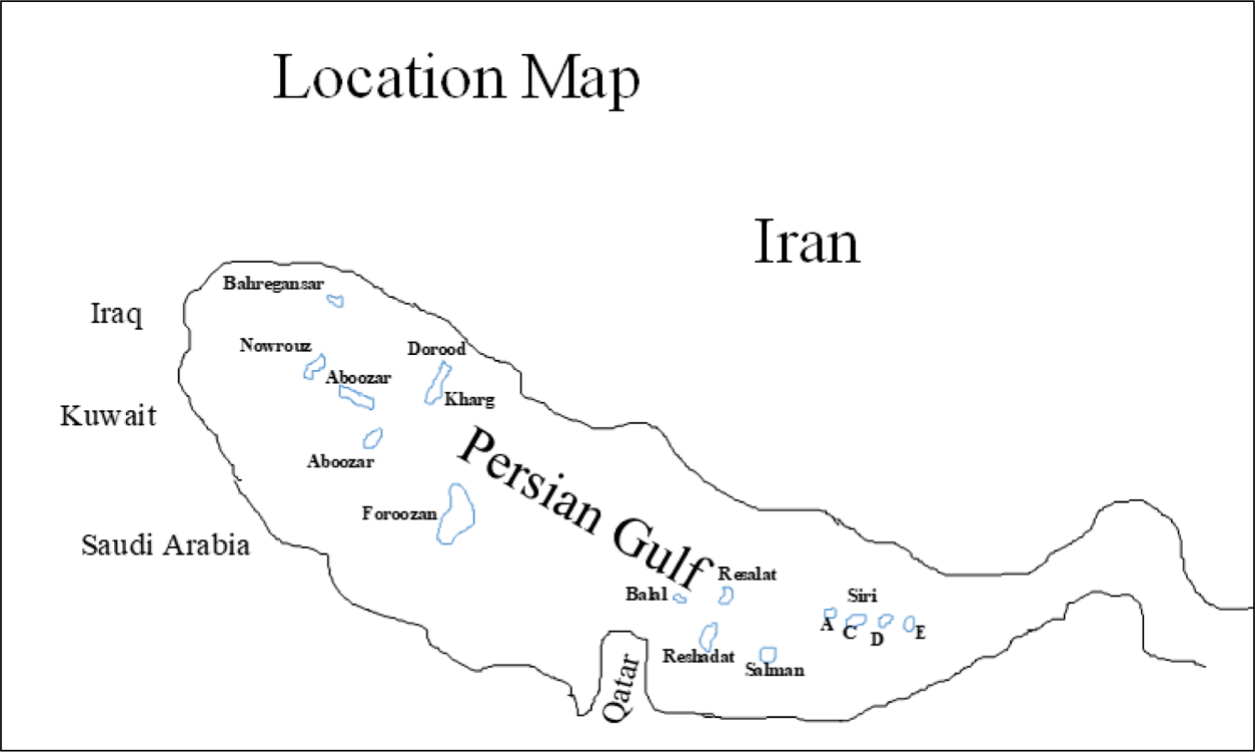
Geographical map of the studied oil fields.

Collectively, 60 oil samples were collected from the literature.^2,3,37^ These samples belong to different oilfields in the Iranian part of the Persian Gulf. This Gulf and its coastal regions are home to about two-thirds of the world’s proven oil reserves (715 billion barrels).^38^ The examined oilfields include Dorood, Kharg, Aboozar, Foroozan, Salman, Resalat, Reshadat, Balal, Bahregansar, Soroush, Nowrouz, Sirri A, Sirri C, Sirri D, and Sirri E. The location map of the studied oil fields is given in Figure 1. Also, the detailed geochemical and biomarker analysis of the studied crude oil samples can be found in Hosseiny et al.,^3^ Mashhadi and Rabbani,^37^ and Rabbani et al.^2^Table 1 summarizes the 16 geochemical and biomarker parameters used as inputs for the SOM network.

**Table 1:** Biomarker Parameters Used as Inputs for the SOM Network and Identified Oil Families^a^

oilfield	% C_27_ steran	% C_28_ steran	% C_29_ steran	steranes/terpanes	C_19_t/C_23_t	C_22_t/C_21_t	C_24_t/C_23_t	C_26_t/C_25_t	C_24_Tet/C_23_t	C_28_BNH/C_30_H	C_29_H/C_30_H	C_30_DiaH/C_30_H	Gam/C_31_HR	C_35_H/C_34_H	d^13^CSAT (‰)	d^13^CARO (‰)	family
Foroozan	39.01	22.15	38.84	0.15	0.22	1.02	0.26	0.43	1.44	0.02	1.34	0.01	0	1.17	–27.1	–27.2	II
Foroozan	41.84	21.32	36.83	0.17	0.25	0.94	0.29	0.48	1.37	0.02	1.36	0.03	0	1.15	–27.2	–27.2	II
Foroozan	36.19	22.35	41.46	0.14	0.25	0.92	0.28	0.43	1.3	0.02	1.42	0.01	0	1.02	–27.3	–27.1	I
Foroozan	42.75	16.33	40.92	0.13	0.24	1.04	0.3	0.46	1.31	0.03	1.52	0.01	0	0.8	–27	–26.7	II
Foroozan	40.7	18.42	40.88	0.16	0.16	1.1	0.39	0.16	1.29	0.02	1.36	0.01	0	0.67	–27.4	–27.2	II
Kharg	40.01	22.59	37.4	0.19	0.21	0.77	0.29	0.43	1.42	0.02	1.4	0.01	0.13	1.12	–27.3	–27	II
Kharg	41.39	20.23	38.38	0.22	0.23	0.83	0.32	0.4	1.52	0.02	1.48	0.01	0	0.91	–27.3	–26.9	II
Kharg	33.24	29.06	37.7	0.34	0.19	0.74	0.39	0.4	1.26	0.03	1.21	0.02	0.24	0.83	–27.3	–27.1	IV
Dorood	38.1	21.26	40.64	0.16	0.21	0.92	0.3	0.39	1.49	0.02	1.46	0.01	0	0.9	–27.4	–27.2	II
Dorood	42.6	22.63	34.77	0.16	0.17	1.09	0.25	0.38	1.51	0.01	1.53	0.01	0.04	1.01	–27.4	–27.2	II
Dorood	31	29.78	40.3	0.35	0.14	0.56	0.56	0.42	1.08	0.04	1	0.03	0.24	0.83	–27.3	–27	IV
Aboozar	33.17	25.18	41.65	0.24	0.18	0.69	0.41	0.41	1.49	0.02	1.14	0.01	0	0.83	–27.7	–27.4	I
Aboozar	34.33	28.7	36.97	0.3	0.13	0.35	0.56	0.48	1.09	0.04	0.96	0.09	0	0.69	–28.5	–27.1	IV
Balal	43.2	22	34.81	0.3	0.83	0.42	0.53	0.5	2.22	0.07	1	0.23	0.25	0.65	–27.3	–26.6	II
Balal	34.51	20.45	45.04	0.16	0.3	0.75	0.37	0.48	1.65	0.02	1.03	0.02	0.1	1.04	–27.1	–26.8	I
Balal	32.3	21.1	46.7	0.15	0.25	0.65	0.43	0.47	1.85	0.02	0.45	0.05	0.14	1.01	–27.5	–27.2	I
Balal	38.3	22	39.7	0.3	0.86	0.34	0.61	0.63	2.55	0.09	0.98	0.12	0.53	0.64	–27.39	–27.08	II
Balal	36.3	23.2	40.5	0.3	0.78	0.39	0.61	0.53	2.51	0.06	1.07	0.15	0.19	0.63	–27.66	–27.04	II
Resalat	36.21	21.08	42.71	0.2	0.39	0.72	0.38	0.4	1.75	0.02	1.03	0.05	0	0.93	–27.2	–26.5	I
Resalat	35.36	19.59	45.05	0.19	0.58	0.65	0.46	0.37	1.89	0.04	1.21	0.04	0	0.97	–27.1	–26.6	IV
Resalat	39.55	26.21	34.25	0.23	0.04	0.97	0.29	0.39	0.34	0.04	0.98	0.01	0.14	1.03	–27	–26.4	IV
Resalat	37.9	29.1	33.1	0.21	0.04	1.08	0.28	0.4	0.36	0.03	1.11	0	0.32	1.35	27.2	26.5	IV
Resalat	31.9	22.2	45.9	0.18	0.47	0.8	0.42	0.44	2.07	0.01	0.92	0.03	0.29	0.97	27.2	–26.6	III
Resalat	33	21.3	45.7	0.11	0.37	1	0.32	0.69	2.01	0.04	1.2	0.09	0.22	0.97	–26.3	–26	I
Resalat	34.38	22.85	42.76	0.14	0.38	0.95	0.29	0.65	1.89	0.04	1.21	0.04	0	0.97	–26.3	–26	I
Reshadat	35.36	19.59	45.05	0.19	0.58	0.65	0.46	0.37	2.08	0.03	0.9	0.06	0	0.85	–27.1	–26.6	I
Reshadat	35.08	29.91	35.01	0.25	0.1	0.58	0.43	0.43	0.58	0.03	0.93	0.02	0.14	0.86	–27.3	–26.2	IV
Reshadat	36.2	28.9	34.8	0.21	0.08	0.57	0.28	0.43	0.59	0.03	0.95	0	0.32	0.91	–27.2	–26.5	III
Reshadat	33	21.9	45.1	0.2	0.53	0.56	0.45	0.36	2.3	0.02	0.98	0.04	0.33	1.08	–27.6	–26.9	I
Salman	34.24	23.34	42.41	0.25	0.55	0.62	0.46	0.45	2.03	0.03	1.03	0.05	0	0.97	–27.2	–26.3	I
Salman	31.13	21.75	47.12	0.24	0.56	0.57	0.47	0.48	2.06	0.03	1.06	0.06	0.12	0.81	–27.2	–26.4	I
Salman	30.43	16.41	53.16	0.17	0.35	0.79	0.66	0.59	1.74	0.02	0.97	0.04	0	0.62	–27.1	–26.7	I
Salman	34.8	20.4	44.8	0.17	0.33	0.8	0.38	0.46	1.89	0.01	1.03	0.03	0.12	0.99	–27.3	–26.8	I
Salman	35.1	25	39.9	0.23	0.35	0.9	0.37	0.42	1.43	0.01	1	0.03	0.15	1	–27	–26.7	I
Salman	37.4	21.4	41.2	0.21	0.45	0.82	0.44	0.5	2.21	0.01	1.02	0.05	0.28	0.97	–27.3	–26.7	II
Salman	34.5	19.7	45.9	0.16	0.34	0.64	0.4	0.41	2	0.02	1.06	0.03	0.09	1.02	–27.4	–26.7	I
Salman	40	20	40	0.26	0.55	0.72	0.49	0.51	2.17	0.01	0.92	0.06	0.13	0.94	–27.4	–26.8	II
Bahregansar	28.83	24.75	46.42	0.33	0.24	0.57	0.46	0.67	1.25	0	1	0.03	0.13	1.2	–27.34	–27.08	I
Bahregansar	34.47	30.73	34.8	0.36	0.09	0.41	0.65	0.81	0.53	0	0.68	0.05	0.23	0.97	–28.21	–27.06	IV
Nowrouz	40	20	41	0.14	0.17	1.1	0.26	0.77	1.66	0	1.18	0.01	0.15	1.22	–27.87	–27.54	II
Soroush	30.46	20.98	48.56	0.22	0.13	0.73	0.36	0.8	1.29	0	1.07	0.03	0.16	1.15	–28.11	–27.6	I
Hendijan	35	27	38	0.57	0.1	0.39	0.74	0.74	0.53	0	0.68	0.05	0.23	0.97	–28.33	–27.08	III
Sirri D	34.56	31.52	33.92	0.24	0.07	0.58	0.4	0.43	0.56	0.04	0.86	0.02	0.09	0.97	–27.1	–26.2	IV
Sirri D	32	31.9	36.1	0.3	0.14	0.54	0.47	0.24	0.53	0.07	0.85	0.02	0.15	0.92	–27.1	–26.3	IV
Sirri D	37	30	33	0.27	0.07	0.67	0.41	0.54	0.51	0.04	0.76	0.02	0.22	0.95	–27.3	–26.3	III
Nousrat	34.54	30.11	35.34	0.24	0.09	0.57	0.44	0.42	0.55	0.04	0.94	0.02	0.08	0.86	–27.1	–26.5	IV
Nousrat	37	29.9	33.1	0.21	0.07	0.67	0.37	0.44	0.52	0.03	0.91	0.02	0.26	1.27	–27.3	–26.1	III
Nousrat	40	29	31	0.22	0.07	0.87	0.36	0.58	0.37	0.04	0.96	0.02	0.24	0.96	–26.9	–26.3	III
Nousrat	34.54	30.11	35.34	0.24	0.09	0.57	0.44	0.42	0.55	0.04	0.94	0.02	0.08	0.86	–27.1	–26.5	IV
Sirri E	36.15	31.9	31.96	0.22	0.1	0.49	0.43	0.44	0.6	0.03	0.89	0.01	0.06	1.1	–27.2	–26.2	IV
Sirri E	38.1	31.1	30.8	0.21	0.1	0.48	0.45	0.39	0.67	0.04	0.85	0.02	0.25	1.28	–27.4	–26.1	III
Sirri E	37.2	31.6	31.3	0.23	0.1	0.49	0.46	0.38	0.67	0.05	0.79	0.02	0.28	1.27	–27.3	–26	IV
Sirri E	32.5	34	33.6	0.27	0.14	0.42	0.5	0.23	0.63	0.02	0.78	0.02	0.25	1.18	–27.1	–26.4	IV
Sirri E	34.5	34	31.5	0.25	0.14	0.42	0.5	0.35	0.59	0.02	0.85	0.02	0.25	1.14	–26.5	–26.6	IV
Sirri A	33.78	31.62	34.6	0.26	0.13	0.47	0.47	0.4	0.66	0.05	0.78	0.02	0.12	0.12	–27.1	–26	IV
Sirri A	35.6	31.6	32.8	0.26	0.11	0.47	0.51	0.46	0.78	0.05	0.77	0.02	0.26	1.16	–27.1	–26.2	IV
Sirri A	36.9	30.1	33	0.24	0.12	0.4	0.5	0.48	0.75	0.05	0.83	0.02	0.3	1.18	–27.2	–26.1	III
Sirri A	35	32	33	0.26	0.11	0.48	0.48	0.53	0.55	0.05	0.84	0.05	0.3	1.17	–27	–26.3	IV
Sirri C	34.39	31.51	34.1	0.25	0.09	0.56	0.41	0.39	0.57	0.04	0.84	0.02	0.11	1.38	–27.1	–25.9	IV
Sirri C	35	31	34	0.25	0.09	0.51	0.46	0.43	0.66	0.04	0.86	0.02	0.27	1.17	–27.3	–26.2	IV
Sirri C	39	27	34	0.26	0.06	0.58	0.43	0.6	0.48	0.04	0.73	0.02	0.25	1.29	–27.4	–26.2	III
Sirri C	37	29	34	0.25	0.08	0.72	0.4	0.57	0.45	0.03	0.77	0.02	0.2	0.9	–27	–26.4	III

a C_19_t/C_23_t: C_19_ tricyclic terpanes/C_23_ tricyclic terpanes, C_22_t/C_21_t: C_22_ tricyclic terpanes/C_21_ tricyclic terpanes, C_24_t/C_23_t: C_24_ tricyclic terpanes/C_23_ tricyclic terpanes, C_26_t/C_25_t: C_26_ tricyclic terpanes/C_25_ tricyclic terpanes, C_24_Tet/C_23_t: C_24_ tetracyclic terpanes/C_23_ tricyclic terpanes, C_28_BNH/C_30_H: C_28_Bisnorhopane/C_30_Hopane, C_29_H/C_30_H: C_29_Hopane/C_30_Hopane, C_30_DiaH/C_30_H: C_30_DiaHopane/C_30_Hopane, Gam/C_31_HR: gamacerane/C_31_HopaneRatio, C_35_H/C_34_H: C_35_Hopane/C_34_Hopane, d^13^CSAT (‰): d^13^CSaturate (‰), d^13^CARO(‰): d^13^CAromaric(‰).

### 2.1 Principal Component Analysis

The first stage in this study was using PCA to decrease the data dimensions. Since 16 different geochemical and biomarker parameters were implemented as inputs, it was mandatory to diminish dimensions to illustrate the data and provide graph results.^39,40^ Accordingly, the data dimensions or components were decreased from 16 to 3 using PCA.

### 2.2 Creating the SOM Network

ANNs mimic the learning process in the human brain. A key component in processing a neural network is the neurons that receive the inputs and generate the outputs using nonlinear operations. The SOM ANN can obtain complex and high-dimension data and extract a visible cluster set.^34^ The process of SOM network training consists of two repetitive phases. The first phase selects the best mapping unit (neural network neurons) to adapt to input data. The second phase is to update the mapping to provide the best representation and display input data.^41^

The process of selecting the best unit to conform to the input data (best matching unit or BMU) is based on the minimum distance (usually the Euclidean distance). Then, in the update phase, each BMU and its neighboring units (within a given radius) move closer to the input data and fully comply with it. This neighborhood radius decreases with each phase selected and updated, eventually leading to a final (two-dimensional) mapping.^42^

The SOM network is composed of an input layer of nodes and an output layer of neurons, in which the grouping of the inputs is formed.^43^ The output layer is called the competitive layer because the competitive role of the network during the training process takes place at this layer. A competitive layer is a two-dimensional plane structured with m neurons while accommodating an input of n neurons. Each input layer neuron with different weight values is connected to the competing layer neurons, and also, a series of minor connections are made between the competing layer neurons.^44^ The number of neurons may vary from a few tens to a few thousands. Each neuron is assigned a dimensional vector d with weight m, of which d is the same dimension as the input vectors. Neurons are connected to their neighboring neurons by a neighborhood relationship that affects the topology or structure of the map. Common topologies are square, hexagonal, triangular, or irregular grids.^45^

**Figure 2:**
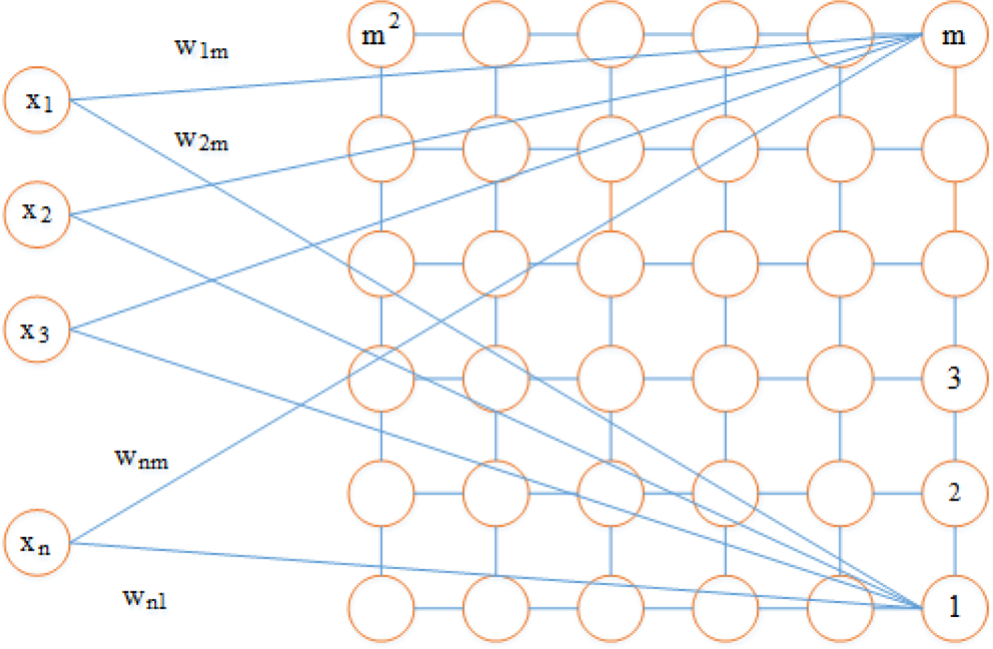
Main structure of an SOM neural network.

As depicted in Figure 2, the SOM neural network consists of a set of *M* = *m* × *m* processing neurons. Suppose these *M* neurons are organized on a grid in a plane. In that case, the obtained network is two-dimensional because this network projects multi-dimensional input vectors onto a two-dimensional surface; for a given network, the input vector x is composed of a fixed dimension n. In the array, the n components of the input vector *x* (i.e., *x*_1_, *x*_2_,..., *x*_*n*_) are connected to each neuron. For a connection from the *i*th component of the input vector to the *j*th neuron, a synaptic weight *w*_*ij*_ is assigned. Thus, an *n*-dimensional vector *w*_*j*_ of synaptic weights is related to each neuron *j*.^46^

In brief, the process of the SOM network is as follows:^46^

(1) Calculate the distance between the pattern (*X*) and all neural neurons^46^
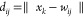
1
(2) Select the nearest neuron as the winning neuron^46^
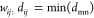
2
(3) Update each neuron according to the neighborhood function.^46^
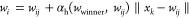
3

The value of coefficient a reduces the effect of different weights.^46^

This process is repeated until a specific stopping criterion is reached. Often the criterion for stopping is a certain number of repetitions. To stabilize the convergence and stability of the map, the learning rate and neighborhood radius are reduced in each iteration. Therefore, convergence will tend to zero. The measuring distance between the vectors is the Euclidean distance.^46^

### 2.3 Clustering Validity Indexes

The clustering validity indexes commonly are used associated with a clustering algorithm. According to the selected index, to determine the exact number of clusters, either minimum or maximum index value aids to figure out the optimum number of clusters (*k*).^47^

Generally, validity indexes can be grouped into internal and external. Internal indexes employ the information related to the data themselves, while external indexes, such as labels, are implemented by external information. Internal measures can improve the clustering algorithms. By contrast, external measures can be used merely for validation. Internal indexes are generally employed to determine the *k* value.^48−51^

In this paper, for the SOM neural network, three efficient internal coefficients, including Davies–Bouldin (DB), Calinski–Harabasz (CH), and Silhouette (SH), were implemented to determine the optimum number of clusters for oil samples. Initially, a number of 10 classes were selected for the SOM network. The model was developed based on these clusters; then, the optimum number of classes as the optimum number of oil families was recognized using the coefficients.

#### 2.3.1 DB Index

This index aims to minimize the average distance between each cluster and the most similar one. The minimum value for the DB index indicates the optimum number of clusters or oil families.^52^

This index is described as^52^
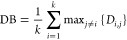
4

in which *D*_*i*,*j*_ shows the within-to-between cluster distance ratio for the *i*th and *j*th clusters. *D*_*i*,*j*_ can be defined as^52^
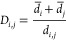
5where *d*_*i*_ represents the mean distance between each point in the *i*th cluster and the cluster’s centroid and *d*_*i*,*j*_ denotes the Euclidean distance between the centroids of the *i*th and *j*th clusters. The optimum clustering solution possesses the lowest DB index value.^52^

#### 2.3.2 CH Index

The CH index^53^ demonstrates the quality of the clustering solution based on the average sum of squares between clusters and within a cluster. It can be measured as^47^
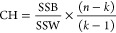
6

in which SSB shows the average between-clusters sum of squares. SSW indicates the average within-cluster sum of squares, *k* represents the number of clusters, and *n* denotes the number of observations. The average SSB is calculated as below^47^
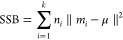
7where *m*_*i*_ is the centroid of cluster I, μ shows the mean of all data points, and ∥*m*_*i*_ – μ∥ typifies the Euclidean distance between the centroid of the cluster and the mean of all data points. The formulation of mean SSW is computed as below^47^
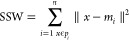
8

in which *k* indicates the number of clusters, *x* is a sample, *p*_*i*_ demonstrates the *i*th cluster, *m*_*i*_ shows the centroid of the cluster *p*_*i*_, and ∥*x* – *m*_*i*_∥ is the Euclidean distance between the sample and centroid of the cluster.^47^

A higher CH quantity epitomizes a better data clustering outcome or the optimum number of questionable clusters. Therefore, high SSB and low SSW numbers give a well-separated cluster.^47^

#### 2.3.3 SH Index

The SH index^54^ demonstrates how close every data point is to other data points within a cluster and how well clusters are detached from each other. Simply put, it operates based on the distance between each point between and within clusters. The highest SH quantity indicates the optimum number of clusters (*k*).^55^
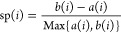
9

in which sp(*i*) is named the silhouette width of a point. *a*(*i*) shows the mean distance between the *i*th point and all the points in the clusters *Pi*, (*i* = 1, 2, ..., *n*). *b*(*i*) displays the most minor of these distances. Hence, it can be observed that the SH value will be between 1 and −1. For every clustering, the average index of all sp(*i*) is employed.^47^ The detailed feature of the SOM network used for clustering in the present study is given in Table 2.

**Table 2:** Features Selected for the SOM Network

topology	distance	CoverSteps	InitNeighbor
Hextop	Linkdist	100	1

## 3 Results and Discussion

Ten clusters as the default numbers have been defined for the SOM network as the definite number of clusters or oil families is unknown. The samples were distributed in these clusters. Nevertheless, the principal objective of this study is to find the optimum number of clusters and hence oil families among these defined clusters. Therefore, validity indexes were employed.

**Figure 3:**
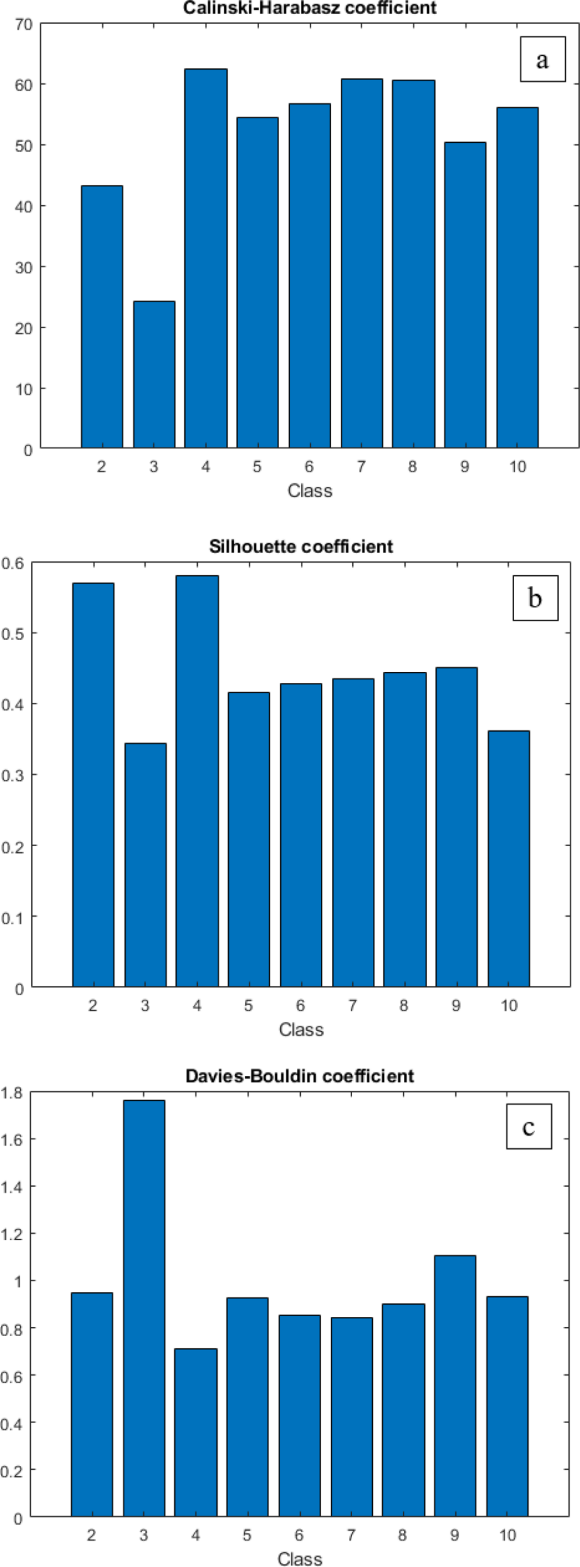
Outcomes obtained by CH (a), SH (b), and DB coefficients (c) demonstrating the optimum number of clusters.

**Figure 4:**
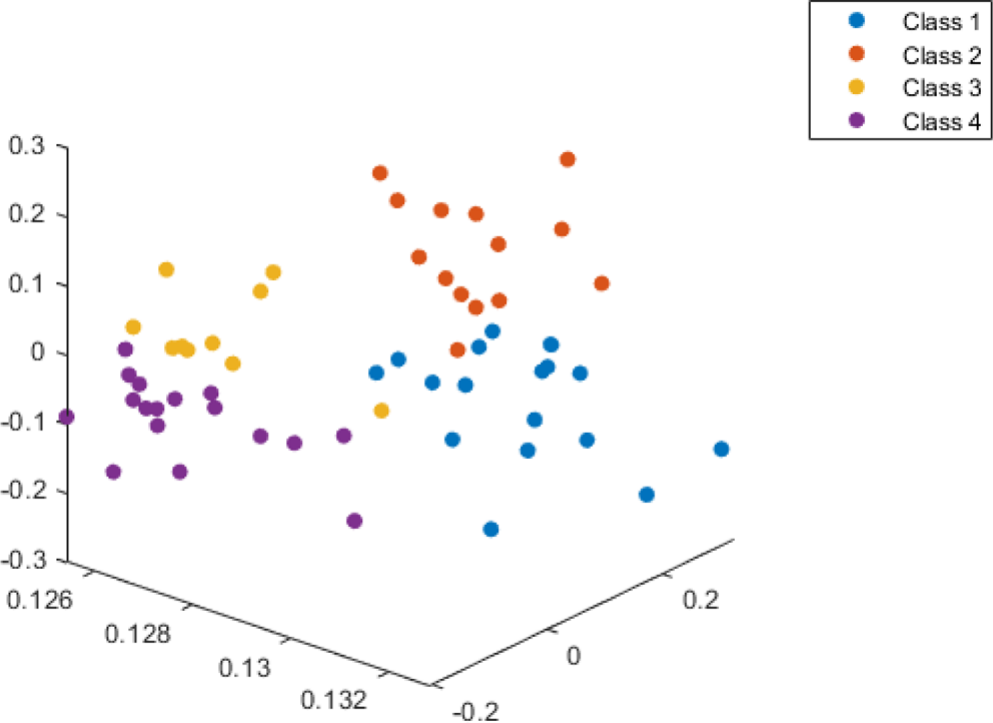
Schematic of the SOM clustering results illustrating four oil families.

Regarding clustering validity coefficients, the maximum values of CH (62) and SH (58) parameters were determined for four clusters (Figure 3a,b). Additionally, the minimum DB coefficient (0.8) was achieved for four clusters (Figure 3c). This means that all three used clustering validity indexes showed four clusters as the optimum number of clusters. Figure 4 in a 3-D shape shows four clusters identified by the SOM neural network. Therefore, it can be concluded that four oil families exist in the Iranian part of the Persian Gulf. In other words, at least four different source rocks have generated the reservoir oils.

Based on the SOM network’s obtained result, cluster I consists of crude oil samples from Foroozan, Aboozar, Balal, Resalat, Reshadat, Salman, Bahregansar, and Soroush oilfields. Cluster II is composed of crude oils from Foroozan, Kharg, Dorood, Balal, Salman, and Nowrouz oilfields. Cluster III contains oil samples from Resalat, Reshadat, Hendijan, Nousrat, Siri A, Siri C, Siri D, and Siri E oilfields. Finally, crude oil samples from Kharg, Dorood, Aboozar, Reshadat, Bahregansar, Nousrat, Sirri A, Sirri C, Sirri D, and Sirri E were grouped into cluster IV.

According to the geochemical parameters summarized in Table 1 and obtained clustering outcomes, C_26_/C_25_ and C_19_/C_23_ tricyclic terpane ratio values lower than 1 for all families suggest a marine depositional environment for the corresponding source rocks. However, the lowest C_26_/C_25_ ratio values for oil family IV (mean = 0.42) suggest a higher water depth during its corresponding source rock deposition. Additionally, family IV signifies a minimum C_19_/C_23_ tricyclic terpane ratio (mean = 0.11), which further supports the higher water depth during the source rock deposition that generated the crude oils of family IV.^56−58^ Furthermore, the mean C_29_/C_30_ hopane ratio values for crude oils in all oil families are greater than 0.8, indicating carbonate lithology for their source rocks.^59,60^ However, higher mean values of this ratio for oil family II may indicate a higher carbonate content in its corresponding source rock. This deduction can be further supported by tricyclic terpanes C_22_/C_21_ and C_24_/C_23_. To explain more, higher C_22_/C_21_ tricyclic terpanes and lower C_24_/C_23_ tricyclic terpanes for crude oils of family II than those of other families could suggest a higher carbonate content in the source rock that has generated crude oils of this family.^1^

Additionally, crude oils of all four families demonstrate C_35_/C_34_ homohopane ratios near unity. Accordingly, a disoxic–anoxic depositional environment can be inferred for the source rocks of all four oil families. Oil family III clarifies a relatively higher mean C_35_/C_34_ homohopane ratio (1.06) than that of other families, and hence, more anoxic conditions can be inferred for the source rock of oil family III.^61^ Also, oil family III depicts a higher gamacerane/C_31_ hopane ratio (mean = 0.25); correspondingly, higher water salinity and a stratified water column can be suggested during the deposition of the source rock of oil family III.^1,62,63^

It is worth mentioning that some crude oil samples have been grouped into more than one oil family. For instance, some crude oils from Balal oilfield have been incorporated into oil family I, and some others have been clustered into oil family II (Table 1). Similarly, several crude oil samples from the Resalat oilfield have been recognized as oil family I, whereas some samples have been known as oil family IV (Table 1). This may suggest the relatively similar geochemical characteristics of the source rocks that have generated the crude oils of different oil families. In previous paragraphs, it was suggested that, generally, source rocks of four identified oil families have been deposited in a similar depositional environment, even though there are some differences.

Overall, the SOM ANN employed in the present paper grouped crude oil samples into four clusters and demonstrated four oil families in the studied area. Hanifa-Tuwaiq, Garau, Diyab member of Surmeh Formation, Kazhdumi, Sarvak, Khatiya, and Ahmadi member of Sarvak formation are regarded as the possible source rocks in the region.^2^ The identified number of oil families is consistent with those suggested by Rabbani et al.^2^ Nonetheless, only 33 samples were analyzed in the mentioned research. However, 60 crude oil samples were analyzed to identify oil families in the present paper to reach more reliable results.

## 4 Conclusions

Lack of novelty in previous studies was the main reason for which we decided to find a new method for identifying oil families, a vital study, in petroleum basins. Thus, an SOM neural network was selected for this purpose. In creating the SOM network, 10 clusters were initially defined in the network. Then, three effective clustering validity coefficients were implemented to identify the optimum number of clusters based on geochemical and biomarker characteristics of oil samples used as inputs for the network. The maximum CH and SH coefficients were acquired for four clusters. Similarly, the lowest DB coefficient was obtained for 4 clusters among 10 defined clusters. Accordingly, all three validation indexes introduced four clusters as the optimum number, hence the number of oil families. Generally, based on the geochemical data, it can be inferred that the source rocks of four oil families have been deposited in a marine carbonate depositional setting with dysoxic–anoxic conditions, although the oil families showed some differences based on geochemical data. Finally, it should be noted that, while some statistical methods such as PCA or HCA can be employed for oil family typing, these approaches have become over-used, and petroleum geochemistry studies and specifically oil family grouping demands novel paradigms. Accordingly, this paper introduced the SOM ANN as a quick and easy-to-use method, which could be greatly beneficial for geochemists in the petroleum geochemistry studies for classification purposes.

## Abbreviations

PCA: principal component analysis
HCA: hierarchical clustering analysis
ANNs: artificial neural networks
CH: Calinski–Harabasz
DB: Davies–Bouldin
SH: Silhouette
AI: artificial intelligence
ML: machine learning
